# Habitat formation prevails over predation in influencing fouling communities

**DOI:** 10.1002/ece3.3654

**Published:** 2017-11-30

**Authors:** Jean‐Charles Leclerc, Frédérique Viard

**Affiliations:** ^1^ UMR 7144 AD2M, Station Biologique de Roscoff Sorbonne Universités, UPMC Univ Paris 06, CNRS Roscoff France; ^2^ Departamento de Ecología Facultad de Ciencias CIBAS Universidad Católica de la Santísima Concepción Concepción Chile

**Keywords:** biotic resistance, facilitation, marinas, marine invertebrates, nonindigenous species, predation, urban ecology

## Abstract

Coastal human‐made structures, such as marinas and harbors, are expanding worldwide. Species assemblages described from these artificial habitats are novel relative to natural reefs, particularly in terms of the abundance of nonindigenous species (NIS). Although these fouling assemblages are clearly distinctive, the ecosystem functioning and species interactions taking place there are little understood. For instance, large predators may influence the fouling community development either directly (feeding on sessile fauna) or indirectly (feeding on small predators associated with these assemblages). In addition, by providing refuges, habitat complexity may modify the outcome of species interactions and the extent of biotic resistance (e.g., by increasing the abundance of niche‐specific competitors and predators of NIS). Using experimental settlement panels deployed in the field for 2.5 months, we tested the influence of predation (i.e., caging experiment), artificial structural complexity (i.e., mimics of turf‐forming species), and their interactions (i.e., refuge effects) on the development of sessile and mobile fauna in two marinas. In addition, we tested the role of biotic complexity—arising from the habitat‐forming species that grew on the panels during the trial—on the richness and abundance of mobile fauna. The effect of predation and artificial habitat complexity was negligible, regardless of assemblage status (i.e., native, cryptogenic, and nonindigenous). Conversely, habitat‐forming species and associated epibionts, responsible for biotic complexity, had a significant effect on mobile invertebrates (richness, abundance, and community structure). In particular, the richness and abundance of mobile NIS were positively affected by biotic complexity, with site‐dependent relationships. Altogether, our results indicate that biotic complexity prevails over artificial habitat complexity in determining the distribution of mobile species under low predation pressure. Facilitation of native and non‐native species thus seems to act upon diversity and community development: This process deserves further consideration in models of biotic resistance to invasion in urban marine habitats.

## 1 INTRODUCTION

Worldwide, coastal hardening is increasing as protection against environmental perturbations (erosion, rising sea level, extreme storms, and flooding; Bulleri & Chapman, 2010; Firth et al., 2016) and to support the development of a diverse set of human activities (shipping, urban development, aquaculture, energy extraction, recreation). Coastal urbanization dramatically influences species diversity and ecosystem functioning across multiple spatial scales, but has nevertheless received less attention than its terrestrial counterpart (Bulleri, 2006; Bulleri & Chapman, 2010).

Adding artificial structures along natural shores is directly associated with habitat degradation, fragmentation, and loss, as well as alteration of connectivity and the local species pool (Bishop et al., 2017; Dafforn et al., 2015). These structures provide novel habitats for colonization by various species, but are not “surrogates” of the neighboring natural rocky reefs (e.g., Connell, 2001b; Fauvelot, Bertozzi, Costantini, Airoldi, & Abbiati, 2009). One specificity of their species assemblages is the high prevalence of nonindigenous species (NIS) compared with natural reefs (Airoldi, Turon, Perkol‐Finkel, & Rius, 2015; Mineur et al., 2012). Although these original urban assemblages probably involve novel ecological interactions, the ecological processes maintaining and acting upon these assemblages are still to be elucidated (Chapman & Underwood, 2011). A growing body of evidence suggests that both the direction and intensity of interspecific interactions such as competition, predation (including grazing), and facilitation (e.g., via habitat formation) can be altered on urban structures compared with observations on and/or expectations for natural habitats (Ferrario, Iveša, Jaklin, Perkol‐Finkel, & Airoldi, 2016; Klein, Underwood, & Chapman, 2011; Rogers, Byrnes, & Stachowicz, 2016; but see Iveša, Chapman, Underwood, & Murphy, 2010).

On intertidal seawalls, which have received much attention, a diverse set of habitat characteristics, such as substrate type, roughness, microhabitats, or slope, can influence community and functional composition (e.g., Chapman & Blockley, 2009; Firth et al., 2016; Moschella et al., 2005). Empirical and experimental studies conducted on these particular artificial structures support ecological predictions linking habitat complexity with species diversity and distribution (Loke & Todd, 2016; MacArthur & MacArthur, 1961; Tews et al., 2004). Increasing habitat complexity of marine artificial structures, for instance by adding pits or grooves in seawalls, has thus been used in ecological engineering projects (see reviews by Dafforn et al., 2015; Firth et al., 2016 and references therein). Taking into account ecological processes (such as habitat–diversity relationships) in the design of artificial marine structures is recommended as a contribution to restoration and reconciliation efforts: Marine artificial structures may provide not only coastal protection but also ecological services (Chapman & Underwood, 2011; Dyson & Yocom, 2015; Evans et al., 2017; Loke, Ladle, Bouma, & Todd, 2015). However, habitat–diversity relationships vary strongly with environmental conditions and local species pools (Loke & Todd, 2016; Matias, 2013). Expectations for a given artificial marine habitat are, to date, uncertain and further comprehensive research is needed to predict which biodiversity and ecosystem functions should be targeted by ecological engineering approaches (Dafforn et al., 2015; Strain et al., in press). Undesirable effects such as the facilitation of NIS are of particular concern (Dafforn, 2017).

Harbors and marinas are perhaps the most invaded habitats of the marine realm (Bax, Williamson, Aguero, Gonzalez, & Geeves, 2003). Rapid spread of NIS occurs in these introduction hotspots and “invasion hubs” (Airoldi et al., 2015; Bishop, Wood, Yunnie, & Griffiths, 2015). They experience strong propagule pressure *s.l*. due to ballast water and hull fouling of cargo ships and leisure craft (Clarke Murray, Pakhomov, & Therriault, 2011; Sylvester et al., 2011). Although environmental conditions are substantially modified in marinas compared with their neighboring habitats (Floerl & Inglis, 2003; Rivero, Dafforn, Coleman, & Johnston, 2013), these “artificial” environments tend to be similar across distant locations, therefore participating in the biotic homogenization of the environment through the establishment of similar NIS communities within and among oceans (Seebens, Gastner, & Blasius, 2013; Streftaris, Zenetos, & Papathanassiou, 2005). The environmental factors that are the most strongly modified in marinas include temperature, salinity, hydrodynamics, sediment resuspension, contaminants, and light (Dafforn et al., 2015). For instance, the network of pilings, pontoons, and poorly sloped seawalls modifies the light environment and contributes to the reduction in cover of habitat‐forming macrophytes (Blockley & Chapman, 2006; Bulleri & Chapman, 2010), either directly, by affecting their recruitment and performance, or indirectly, by favoring the recruitment of epibionts (Marzinelli, Underwood, & Coleman, 2011). Although the reduction or loss of natural seaweed habitats (canopy and understory) may affect associated faunal assemblages, the spatial arrangement of artificial structures creates myriad microhabitats with various structures, properties, and ultimately complexities (Dafforn et al., 2015) that may favor alternative (mostly faunal) habitat‐forming species (Connell, 2001b; Sellheim, Stachowicz, & Coates, 2010), including NIS (Dafforn, 2017).

To date, the role of habitat complexity in artificial coastal habitats has mainly been investigated on fixed intertidal structures, such as seawalls and riprap (e.g., Firth, Browne, Knights, Hawkins, & Nash, 2016; Firth et al., 2016; Martins, Thompson, Neto, Hawkins, & Jenkins, 2010; Moschella et al., 2005). Less attention has been paid to floating subtidal structures such as floating pontoons which differ however in many ways (Holloway & Connell, 2002; but see Lavender, Dafforn, Bishop, & Johnston, 2017). Fouled by unique assemblages, they generally support more abundant and diverse NIS than do fixed structures (Dafforn, 2017; Dafforn, Johnston, & Glasby, 2009; Glasby, Connell, Holloway, & Hewitt, 2007). Owing to their direct proximity and similarity to vessel hulls, floating pontoons are likely to act as important stepping stones involved in the spread of NIS at local scales. These habitats undergo high disturbance due to maintenance work and multiple stressors, such as variation in salinity due to rainfall and exposure to pollutants from adjacent ships, which can give a competitive advantage to tolerant and fast‐growing NIS (Piola & Johnston, 2008; Saloni & Crowe, 2015). Free space being the main limiting resource on simple hard substrata, competition is considered to be one of the most important interactions occurring among sessile species across multiple life cycles (Rius, Potter, Aguirre, & Stachowicz, 2014; Stachowicz, Fried, Osman, & Whitlatch, 2002). As such, competition may play a key role in “biotic resistance” against NIS establishment, as originally formulated (Elton, 1958). As various habitat‐forming species may compose fouling communities (Sellheim et al., 2010), facilitation of both sessile and mobile species, native or exotic, is pervasive and deserves attention (Bulleri, Bruno, & Benedetti‐Cecchi, 2008; Floerl, Pool, & Inglis, 2004).

In these habitats, specific species interactions must be considered. Most floating structures are out of reach for benthic consumers (Dumont, Harris, & Gaymer, 2011; Rogers et al., 2016), and potential top‐down controls are more likely to involve swimming megapredators (generally fish and crustaceans) and mobile macroinvertebrates associated with fouling assemblages (Connell, 2001a; Rogers et al., 2016). The influence of predation *s.l*. (i.e., including grazing) in these habitats is not yet well established and may depend on consumer mobility and size. Some mobile invertebrates (e.g., chitons, amphipods) associated with fouling communities forage on sessile species, especially the early stages of solitary ascidians, thus affecting succession of fouling communities (Nydam & Stachowicz, 2007; Rius et al., 2014). The effects of megapredators (e.g., fish, crabs) are more complex and vary strongly depending on the prey taxa and the spatial scale. For example, in New South Wales, Australia, predation by fish has a weak effect in Sydney Harbour (Connell, 2001a), whereas in Botany Bay, there is a stronger effect (Lavender, Dafforn, & Johnston, 2014) on the abundance of similar taxa. Finally, there is virtually no information on the trophic links between large predators and mobile invertebrates (but see Lavender et al., 2014). In this context, questions remain on the cascading effects which may result from these interactions (Thomsen et al., 2010).

Using an experimental approach, we investigated whether swimming predators influenced the early development of sessile and mobile fauna (hereafter SF and MF, respectively) associated with floating pontoons in marinas. In addition, we tested whether artificial complexity affects the richness, the abundance, and the assemblage structure of SF and MF. More specifically, we predicted that increasing surface area of the initial artificial substrate would mainly facilitate MF colonization, for example, by providing refuges from megapredators, and in turn promote macropredation on SF. To examine this scenario (i.e., that habitat complexity mediates species interactions), complexity and exclusion treatments were crossed with each other. We predicted interaction between these factors, and more particularly that predation on MF by megapredators is higher on simple substrata than on complex substrata, thereby decreasing abundance and modifying assemblage structure. In addition to the effect of the initial artificial habitat complexity, we examined whether biotic complexity (i.e., habitat formation through sessile species development) also enhances the richness and abundance of mobile invertebrates. We were particularly interested in examining the hypothesis that biotic complexity decreases the abundance or richness of NIS, possibly contributing to the biotic resistance to invasion.

## 2 METHODS

### 2.1 Study sites

**Figure 1:**
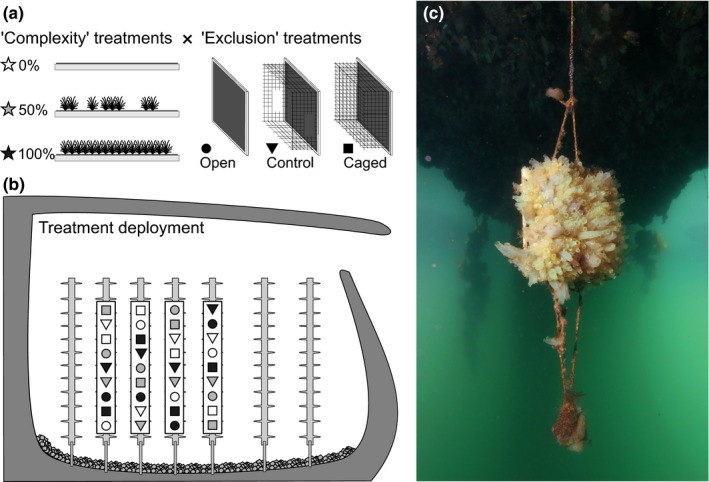
(a) Experimental treatments testing for the effect of “Complexity” (0%, 50%, and 100% of astroturf cover) and “Exclusion” (Open, Cage‐Control, and Caged). (b) Treatments' deployment along floating pontoons in marinas. (c) Example of an “open” panel after 2.5 months (Brest, 08/2014, Courtesy of W. Thomas, SBR)

The study was conducted in two marinas, located 80 km apart, in NW Brittany (France): Trébeurden (48.7700°N, 3.5870°W) and Brest‐Château (hereafter “Brest,” 48.3790°N, 4.4890°W). Both marinas are fully marine and protected from wave action by breakwaters (Figure 1b). The Trébeurden marina is semi‐enclosed and holds water for a few hours during low spring tides (a sill is located at 2 m above chart datum). The Brest marina is deep enough to allow permanent ship movement and is thus permanently open to circulation. Although both marinas are of similar carrying capacity along plastic floating docks (625–650 berths), Brest (140,000 residents) is one of the main hotspots of the military, commercial shipping, and recreational boating along the Atlantic coast of France, whereas Trébeurden (3,700 residents) is likely less affected by human activities (pollutants, maritime traffic) and hosts only leisure craft.

### 2.2 Setup and experimental design

Artificial substrata were composed of a matrix of white, fluted polypropylene panels (3.5 mm thick, 180 × 180 mm). A total of nine treatments were set up based on the two factors tested (“exclusion” and “complexity,” each with three levels) as well as their interactions (Figure 1a).

We examined the effects of predation, here defined by carnivorous, omnivorous, and grazing activities, which can affect the living component of the fouling communities (i.e., foraging on seaweeds, mobile, and sessile fauna). In our study, two categories of predators were examined according to size (e.g., Wei et al., 2011 for delimitation of size classes): macropredators sized >1–2 mm and megapredators sized >10–20 mm.

The effect of megapredators on fouling communities was tested through “exclusion” treatments: open, cage‐control, and caged (Figure 1a). Cages were constructed from plastic fencing (10 × 10 mm mesh (165 × 165 × 100 mm), which surrounded the turf patch (see below) to exclude all megapredators. Cage‐controls were cages lacking a roof and with two windows (40 × 60 mm) cut out on the sides to prevent “behavioral artifacts” of mobile prey, which could seek refuge in cage‐controls (Steele, 1996). Open treatments were panels without cages. The abundance and assemblage structure of mobile megafauna, targeted by the exclusion treatments, were assessed beneath floating pontoons, as detailed in Appendix S1.

To test for the effect of initial habitat complexity, the density of structural elements—a key variable of “habitat complexity” (Kovalenko, Thomaz, & Warfe, 2012)—was manipulated. We used a matrix of commercial artificial grass, hereafter named “turf,” mimicking turf‐forming species. Turf‐forming species include, for instance, coarsely branched, corticated, and jointed calcareous algae (Connell, Foster, & Airoldi, 2014; Littler & Littler, 1984), and sessile invertebrates with prostrate and upright branches being more or less erect and dense, such as erect bryozoans and some hydrozoans. The former were little observed, probably due to severe light attenuation under pontoons, but the latter were commonly found in the study sites (see Section 3). The turf matrix was made of bundles of ca. 30 green polypropylene strips (30 mm long, 2 mm wide) separated by 6 mm and attached on a canvas sheet in regular rows (10 mm apart). To create “complexity” treatments (0%, 50%, or 100% of turf density; Figure 1a), bundles were stripped from the canvas sheet along randomly selected rows (Figure 1a). Artificial turf (165 × 165 mm) was then stapled on the panels (Figure 1a). The canvas sheet (used in all treatments) allowed successful development of fouling assemblages (Figure 1c).

All panels were deployed by snorkelers using ropes and leads, at an intermediate position respective to pontoon width and at a constant depth of 1 m. This shaded position and depth were chosen to facilitate settlement of the targeted fouling invertebrates and to prevent the influence of confounding factors such as die‐off in response to transient variation in salinity at the surface due to rainfall (Bouchemousse, Lévêque, & Viard, 2017; Pineda, Turon, Pérez‐Portela, & López‐Legentil, 2016).

At both sites, 36 panels were deployed: Nine panels (one per treatment) were randomly distributed (3 m apart) along each of four adjacent pontoons (Figure 1b), giving an “unreplicated randomized block design” (Underwood, 1997) or a “randomized complete block design” (Quinn & Keough, 2002). The lack of within‐cell replication (i.e., within each combination of pontoon × complexity × exclusion) prevented testing for in the highest‐order interaction term (Anderson, Gorley, & Clarke, 2008; Underwood, 1997). Nonetheless, the spatial block design makes it possible to attribute part of the total variance to differences between blocks (here “pontoons” corresponding to discrete spatial units within each marina) and thereby reduce the residual unexplained variation (Quinn & Keough, 2002).

The experiment was conducted between 19–20 May and 1–4 August 2014, a season favorable to the settlement of juveniles of many invertebrate species in the study area (Bouchemousse, 2015; Bouchemousse et al., 2017). The experiment duration (11 weeks) allowed diverse and abundant sessile and mobile fauna to colonize the panels (Figure 1c), as well as biotic interactions to take place within the fouling assemblages (Lord & Whitlatch, 2015; Sellheim et al., 2010; Stachowicz et al., 2002). To prevent flow disruption, ropes and cages were cleaned of epibiota every 3 weeks using a plastic brush. By the end of the trial, panels were retrieved by snorkelers using polypropylene rubble bags (mesh <0.5 mm) to minimize mobile fauna (MF) loss. Back in the laboratory, panels were removed from their bags, cleared from cages, and left with all remaining bag contents in seawater tanks until sessile fauna (SF) returned to their natural, untense state. Before preservation (in 3% formaldehyde), photographs were taken to record the natural appearance of SF to facilitate future identification.

### 2.3 Data collection

Before assessing species abundance, the final structural complexity of the microhabitat (panel) was assessed using two parameters: (1) the total SF volume and (2) the interstitial volume left among SF (and turf, when present). When growing on substrates, several sessile species (especially solitary tunicates) tend to occupy the initial interstitial surface (created among turf mimics), and make additional surfaces available for colonization by epibionts. The habitat complexity thus changed over time due to colonization by these habitat‐forming species. To measure these changes, each panel was placed in a thin plastic bag (negligible volume) and plunged in a transparent water jar to measure its total displacement volume. The same procedure was repeated without the plastic bag. The interstitial volume, hereafter named “ecospace” (Jones, 1971), was determined by the difference in water displacement with and without the plastic bag (Leclerc, Riera, Lévêque, & Davoult, 2016). The SF volume was estimated by the difference in water displacement before and after deployment (i.e., between blank—including turfs when present—and colonized panels).

Abundance was assessed for SF and MF using percentage cover and numerical distribution, respectively. To avoid edge effects in SF distribution, a 15 mm perimeter was excluded from analysis, giving a 150 × 150 mm working area. SF cover was estimated by summing over 25 subquadrats, within which a score from 0% to 4% was given to each taxon (Dethier, Graham, Cohen, & Tear, 1993). To take into account species layering, percent cover was assessed for epibiotic, habitat‐forming (e.g., solitary tunicates) and understory species; therefore, the total frequently exceeded 100%. Following SF identification, panels were washed thoroughly with freshwater through a 500‐μm‐mesh sieve to sort MF before identification and counting under a microscope. Both SF and MF specimens were identified at the lowest taxonomic level possible (generally species; Tables S2 and S3) and categorized as “native,” “nonindigenous,” “cryptogenic,” or “unassigned” according to the literature and databases (WORMS/WRIMS; Pagad, Hayes, Katsanevakis, & Costello, 2016). MF were also sorted according to their main function within the food web (carnivores, suspension‐deposit‐feeders, herbivores). The cryptogenic species, from unknown/uncertain origin (sensu Carlton, 1996), found in this study (mainly amphipods and nudibranchs; Table S3) displayed a cosmopolitan distribution and were either European‐native introduced elsewhere or introduced in Europe from an unknown origin. Cryptogenic and NIS, both candidates for to further introduction and spread, were pooled in analyses (Dafforn et al., 2009).

### 2.4 Statistical analyses

Both univariate and multivariate data were examined with a four‐way design using permutational multivariate analysis of variance (PERMANOVA; Anderson et al., 2008) with 4,999 permutations. Factors were Site (random, two levels: Brest and Trébeurden), Pontoon (random, nested within site), Exclusion (fixed, three levels: Open, Cage‐Control, and Caged), and Complexity (fixed, three levels: 0%, 50%, and 100%). Euclidean distance and Bray–Curtis similarity matrices were respectively used for uni‐ and multivariate analyses. Given that panel deployment targeted fauna, the few seaweeds, that is, on average less than 2% of cover (Table S2), found poorly attached (either on panels or on sessile fauna) were not included in data analyses. Combined with multivariate PERMANOVAs, samples were ordinated using principal coordinate (PCO) analyses. The homogeneity in univariate or multivariate dispersion was checked using PERMDISP for all factors (appropriately combined according to Anderson et al., 2008).

To examine whether the richness, abundance, and assemblages of MF were affected by the habitat complexity due to SF, two covariates were incorporated in the design described above: (1) SF volume and (2) ecospace. These two metrics were not correlated (*R *=* *.131, *p *=* *.272) and thus could be used as covariates. The interaction terms between the covariates and the fixed factors were nonsignificant and thus removed from the analyses. Finally, the importance of the spatial arrangement of SF at the scale of the microhabitat (i.e., panel) was further investigated. Distance matrices for cover or similarity matrices for multivariate assemblage structure of SF were compared with distance/similarity matrices computed for MF richness, abundances, and community structure using the RELATE procedure (Clarke & Warwick, 2001). Analyses were conducted using cover data from either habitat‐forming species (solitary ascidians from the genera *Ciona*,* Ascidiella*, and *Styela*) or all SF combined, because epibiota also contribute to structural complexity (Thomsen et al., 2010).

Statistical analyses were performed on either all taxa combined (including unassigned), native taxa, or pooled nonindigenous and cryptogenic species (Thomsen, Wernberg, South, & Schiel, 2016). Separate analyses on trophic groups of MF (including carnivores) were carried out with the same results. Only results of the analyses on all MF are thus presented. All analyses were performed using PRIMER 6 (Clarke & Warwick, 2001).

## 3 RESULTS

### 3.1 Successful panel colonization by abundant and diverse assemblages

**Figure 2:**
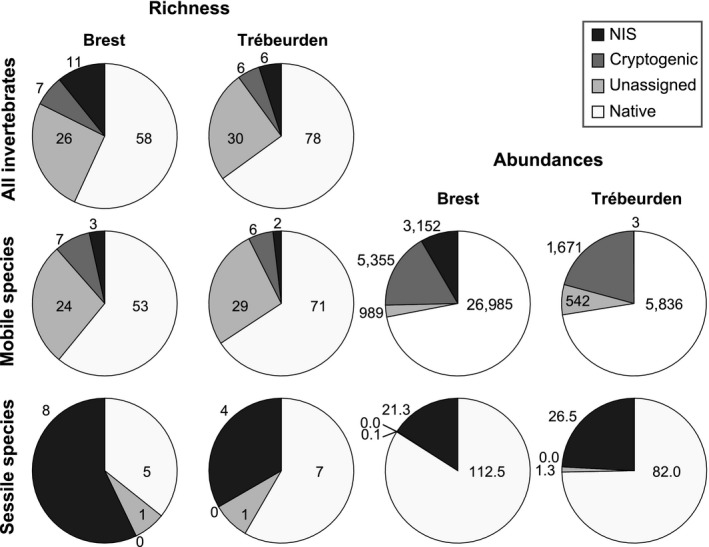
Proportions and absolute richness (depicted values) of nonindigenous, cryptogenic, unassigned, and native species in the study sites for all species, mobile fauna (MF), and sessile fauna (SF). Proportions and absolute abundances are given for mobile (numerical) and sessile (mean cover) species separately

**Figure 3:**
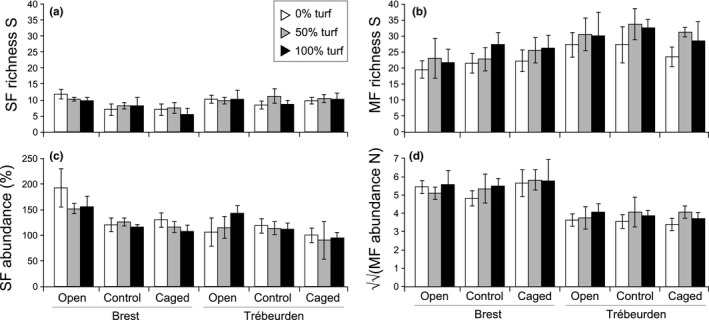
Macrofauna richness (a, b) and abundances (c, d) depicted for sessile fauna (SF; left panels) and for mobile fauna (MF, right panels) observed on panels at the end of the experiment in Brest and Trébeurden across complexity (0%, 50%, and 100% cover of turf) and exclusion (Open, Cage‐Control, and Caged) treatments. SF abundance is expressed as percentage cover (%), while MF abundance is expressed as counts (square‐root‐transformed)

**Figure 4:**
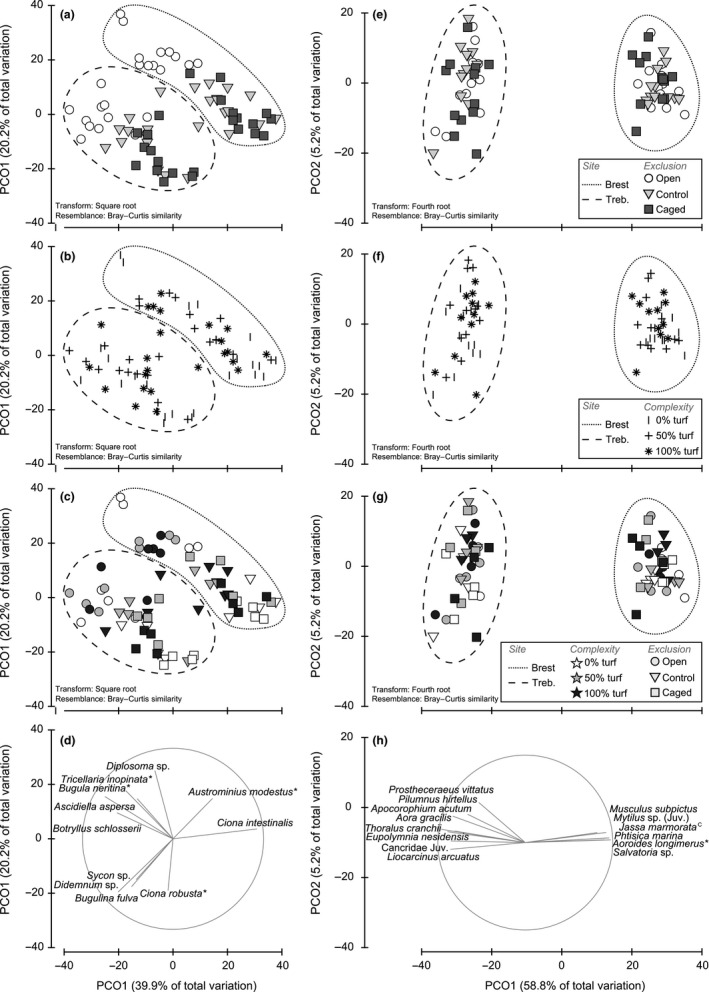
Principal coordinate analyses describing sessile fauna (SF; left panels) and mobile fauna (MF; right panels) assemblage structure. Ordinations are displayed for either Exclusion (a, e), Complexity treatments (b, f), or the interaction term (c, g). Dotted lines show site delineation. Vector plots of variable correlated with the PCO axes are indicated for SF (*r* > .5, d) and MF (*r* >* *.7, h), with * and ^c^ highlighting NIS and cryptogenic species, respectively

Across sites, a total of 165 faunal species were identified corresponding to 21 sessile and 144 mobile invertebrates. Complete lists and species authorities are provided for sessile fauna (SF) and mobile fauna (MF) in Tables S2 and S3, respectively. Despite being at an early stage of succession (2.5 months), the assemblages were diverse (37.7 ± 6.7 species per panel, ±*SD*) and showed very high abundance (SF: 122.6 ± 6.7% cover, MF: 584.2 ± 508.6 individuals). Differences in SF richness, total cover, and assemblage structure were observed between sites (Table 1, Figures 3 and 4), although fouling cover was generally dominated by solitary ascidians (e.g., *Ciona intestinalis*,* Ascidiella aspersa*,* Ciona robusta*). Extensive covers of sheetlike colonial ascidians and bryozoans (e.g., *Diplosoma listerianum*,* Watersipora subatra*) and erect bryozoans (*Bugula neritina, Tricellaria inopinata*) were also recorded (Table S2) either directly on the panel surface or as epibionts of the habitat‐forming ascidians. NIS accounted for 33.3%–57.1% of the richness and 15.9%–24.2% of the SF cover (Figure 2). Like SF, the MF communities differed substantially in terms of richness, abundance, and assemblage structure between sites (Table 2, Figures 3 and 4). They also differed among pontoons within a site, which may reflect different MF assemblages among pontoons to which panels were attached (assuming that MF that colonize panels originate from the pontoon to which the panels were attached; Table 2). Overall, MF were numerically dominated by (1) suspension‐feeders (61.9% in Brest and 53.7% in Trébeurden), in particular tube‐dwelling (*Jassa marmorata*,* Monocorophium acherusicum*) or swimming (*Aoroides longimerus*, in Brest) amphipods and (2) carnivores (37.4%–41.5%) such as grasping caprellids (*Phtisica marina*) and crawling annelids (*Harmothoe impar*,* Syllidia armata*). Herbivores, dominated by tube‐dwelling annelids (*Platynereis dumerilii*) and gastropods (*Rissoa parva*), only accounted for 0.7%–4.8% of the abundance. Pooled together, nonindigenous (e.g., *Aoroides longimerus*) and cryptogenic (*Monocorophium acherusicum*) mobile species accounted for 7.4%–11.5% of the richness and 20.8%–25.4% of the abundance (Figure 2).

**Table 1:** Results of PERMANOVA tests for differences in sessile fauna (SF) richness, abundance (percentage cover), and assemblage structure. Significant multivariate dispersion (PERMDISP) around the centroid among nested and crossed factors is summarized (*p < .05; **p < .01; ***p < .001). Pairwise tests are summarized considering Complexity (0%, 50%, and 100% turf), Exclusion treatments (Op: Open; CC: Cage‐Control; Ca: Caged), and sites (Brest, Trébeurden)

		SF											
		Richness S				Total percentage cover				Assemblage structure			
Transformation		None				None				Square root			
Distance/Similarity		Euclidean distance				Euclidean distance				Bray–Curtis similarity			
Source	*df*	DISP	MS	Pseudo‐*F*	*p*	DISP	MS	Pseudo‐*F*	*p*	DISP	MS	Pseudo‐*F*	*p*
Site = Si	1		43.6	20.364	**.031**		10952	41.521	**.030**		17848	40.657	**.025**
Complexity = Co	2		3.9	2.128	.379		519.26	0.398	.743		2339.8	1.890	.205
Exclusion = Ex	2		26.1	1.083	.568	**	8845.1	3.819	.231		6918.1	4.305	.182
Pontoons = Po (Si)	6		2.1	0.692	.650		263.77	0.639	.695		439	1.021	.438
Si × Co	2		1.8	0.496	.613		1305.9	5.961	**.014**		1237.8	4.729	**.001**
Si × Ex	2		24.1	18.826	**<.001**	*	2316.2	5.777	**.020**	*	1606.9	4.455	**<.001**
Co × Ex	4		4.7	1.659	.329	***	331.56	0.382	.806		713.12	2.335	.122
Po (Si) × Co	12		3.7	1.205	.341		219.09	0.531	.864		261.74	0.609	.980
Po (Si) × Ex	12		1.3	0.413	.946	*	400.91	0.972	.495		360.69	0.839	.777
Si × Co × Ex	4		2.8	0.921	.477		868.54	2.105	.109	***	305.38	0.710	.789
Res	24		3.1				412.54				429.87		
Pairwise tests		Si x Ex	Brest	Op > CC = Ca		Si × Co	Brest	0% > 50% = 100%		Si × Co	Brest	0% ≠ 50% = 100%	
			Treb.	Op = CC = Ca			Treb.	0% = 100% = 50%			Treb.	(0% ≠ 100%) = 50%	
						Si × Ex	Brest	Op > CC = Ca		Si × Ex	Brest	Op ≠ CC = Ca	
							Treb.	Op = CC = Ca			Treb.	Op ≠ CC = Ca	

Significant *p*‐values (< .05) are indicated in bold.

**Table 2:** Results of PERMANOVA tests for differences in species richness, numerical abundance, and abundance distribution of mobile fauna (MF). See abbreviations in Table1

		MF											
		Richness				Numerical abundance				Assemblage structure			
Transformation		None				Fourth root				Fourth root			
Distance/Similarity		Euclidean				Euclidean				Bray–Curtis			
Source	*df*	DISP	MS	Pseudo‐*F*	*p*	DISP	MS	Pseudo‐*F*	*p*	DISP	MS	Pseudo‐*F*	*p*
Ecospace	1		386.6	19.77	**<.001**		2.0	5.13	**.035**		1849.1	2.95	**.001**
SF volume	1		651.8	1.45	.525		23.1	0.57	.712		30398.0	0.97	.531
Site = Si	1		343.8	10.16	**.030**		30.9	62.00	**<.001**	***	23737.0	39.30	**<.001**
Complexity = Co	2		31.6	1.47	.321		0.6	3.10	.141		651.3	0.83	.589
Exclusion = Ex	2		22.8	2.39	.249		0.3	0.70	.564		739.2	1.07	.407
Pontoons = Po(Si)	6		51.4	3.01	**.027**		0.8	4.57	**.004**	***	736.1	1.54	**.011**
Si × Co	2		21.5	1.03	.389		0.1	0.45	.651	***	999.0	2.27	**.004**
Si × Ex	2		6.8	0.27	.764		0.4	1.58	.249	***	710.6	1.35	.179
Co × Ex	4		12.6	0.95	.512		0.4	5.71	.070		476.0	0.79	.729
Po(Si) × Co	12		20.9	1.22	.333		0.3	1.46	.217		434.1	0.91	.731
Po(Si) × Ex	12		26.2	1.54	.188		0.2	1.36	.254	**	523.1	1.10	.271
Si × Co × Ex	4		12.8	0.75	.568		0.1	0.32	.866	**	611.1	1.28	.131
Res	22		17.0				0.2				477.7		
Pairwise tests										Si × Co	Brest	0% = 50% = 100%	
											Treb.	(0% ≠ 100%) = 50%	

### 3.2 Absence of megapredator influence on species assemblages

The mobile megafauna recorded beneath pontoons were dominated by predators, ranging from strictly carnivorous (*Gobiusculus flavescens*,* Palaemon* spp.) to omnivorous species (e.g., *Chelon labrosus*). The total abundance of megafauna found under floating pontoons was similar at the two sites. They shared the most abundant species, including fish, such as *Chelon labrosus*,* Gobiusculus flavescens*, and *Atherina presbyter*; and crustaceans, such as *Palaemon* spp. (Appendix S1). Additional fish species were found abundant (e.g., *Ctenolabrus rupestris*,* Pollachius pollachius, Lipophrys pholis*; Table S2, Figure S2) on nearby riprap in the inner part of the marina.

Overall, neither SF nor MF were affected by megapredators (Figures 3 and 4a,e), whether analyses were performed on all, native, or NIS with cryptogenic species (Tables S4 and S5). A significant Site × Exclusion interaction was found for SF richness, abundance, and community structure (Table 1), but pairwise tests indicated that the open treatments differed from both cage‐control and caged panels. This result was consistent for both response variables in Brest, where open treatments showed greater SF richness and total percentage cover (Figure 3a,c). At this site, cage‐control and caged treatments displayed a high abundance of *Ciona* spp. and low abundance of *Ascidiella aspersa* and associated epibionts such as *Tricellaria inopinata, Bugula neritina, Watersipora subatra, Botryllus schlosseri*, and *Diplosoma* sp. (all explaining ca. 73% of dissimilarity with open treatments, SIMPER; Figure 4d). Although cages were frequently cleaned, the observed caging effect may be due to preferential larval settlement of *Ascidiella aspersa* and its epibionts on the fencing mesh (observed during cleaning) rather than on the panels. It is also possible that additional shading due to the mesh helped *Ciona* spp. to outcompete the other taxa in the caged treatments.

The most important result is that no differences occurred between cage‐control and caged treatments, indicating no megapredation effect. Like for SF, exclusion had no effect on MF (Figure 3b,c,e, Table 2). Additionally, our analyses did not reveal any interaction between “Complexity” and “Exclusion,” either for all SF or for MF. Although such interactions were significant for the native SF richness and the NIS percentage cover (Table S4), inconsistent results were found when examining pairwise tests. For instance, in Brest, higher NIS cover was observed on bare panels (0%) than on turf treatments (50% and 100% turf) in open and caged treatments, but not in cage‐controls. Overall, these results reject the prediction that the artificial initial complexity of the microhabitat influenced megapredator foraging.

### 3.3 Absence of influence of artificial complexity on MF assemblages

Conversely to our expectations, artificial turf had an effect on neither abundance nor MF richness (Figure 3b,c; Table 2), even when considering macropredators only. As for community structure, the sole observed difference was between extreme complexity treatments (0% and 100% turf) in Trébeurden only (Table 2; Site × Complexity). This result was observed for native assemblages, but not for nonindigenous and cryptogenic MF (Table S5). Similarly, the initial microhabitat complexity did not affect SF richness (Table 1, Figure 3a). PERMANOVA indicated a significant effect on SF community structure (probably driven by NIS; Table S4): Bare panels were different from turf treatments (Table 1; Site × Complexity) at both sites and were associated with greater SF cover in Brest. A set of bryozoans (e.g., *Watersipora subatra*,* Tricellaria inopinata*) appeared slightly more abundant on bare treatments (SIMPER). Careful examination of PCO (Figure 3b; 60.1% variation explained), MDS (not shown), and PERMDISP (*F*_2,69_ = 2.91, *p *=* *.072, deviation from centroid; “0%”: 31.0 ± 1.7, “50%”: 28.1 ± 1.4, “100%”: 26.2 ± 1.1) suggests that these differences may partly be explained by the homogenization of community structure with increased turf density (i.e., marginally nonsignificant reduction of multivariate dispersion).

### 3.4 Prevalence of habitat formation in shaping associated assemblages

**Figure 5:**
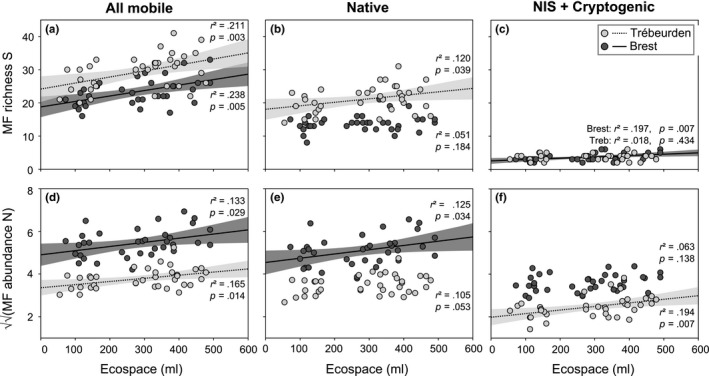
Mobile fauna (MF) richness and abundance variation with interstitial volume (ml) in Brest (pale gray) and Trébeurden (dark gray). Relationships are given for all mobile species (a, d), for native species (b, e), and for nonindigenous (NIS) and cryptogenic species combined (c, f). Only significant linear regressions (*p *<* *.05) are depicted (with 95% CI,* p*, and *r*²)

The total SF volume did not influence MF, but ecospace (i.e., interstitial volume created among SF) had a significant effect on richness, abundances, and community structure of MF (Table 2). In addition, positive relationships were observed between ecospace and both richness and abundance in the two sites (Figure 5a,d), regardless of initial turf complexity.

In addition, the total cover of habitat‐forming ascidians (the species most responsible for ecospace) correlated with the assemblage structure of MF (ρ = .310, *p *<* *.001), although neither with their richness (ρ = .042, *p *=* *.173) nor with their abundance (ρ = .007, *p *=* *.519). The assemblage structure of these habitat‐forming ascidians was also related to MF richness (ρ = .123, *p *=* *.002), abundance (ρ = .103, *p *=* *.023), and assemblage structure (ρ = .317, *p *<* *.001). Even better relationships were observed using the assemblage structure of all SF (with MF richness, ρ = .124, *p *=* *.001; abundance, ρ = .282, *p *<* *.001; and community structure, ρ = .449, *p *<* *.001). Altogether, these results suggest that both spatial arrangement and SF species composition influence MF.

Contrasting results were observed between sites when examining separately each MF category (native, cryptogenic, NIS). Ecospace positively influenced the richness of native species in Trébeurden (Figure 5b) and of nonindigenous and cryptogenic species in Brest (Figure 5c), whereas it favored the numerical abundance of native species in Brest (Figure 5e) and of nonindigenous and cryptogenic species in Trébeurden (Figure 5f). In both sites, NIS were thus influenced by ecospace, but with different patterns.

## 4 DISCUSSION

In both study sites, creating artificial complexity had no effect on the fouling assemblages at an early successional stage or on megapredation, which appeared negligible underneath floating pontoons. Most species interactions occurred within fouling communities, especially through facilitation by habitat formation due to species growing on the substratum.

Various megapredators were encountered underneath the floating pontoons, and some of them were observed feeding on the associated fouling communities (e.g., *Chelon labrosus*). Nevertheless, in both marinas, the complete exclusion of these consumers did not affect the development of sessile assemblages, regardless of species category (i.e., native, cryptogenic, and NIS). These results suggest that the megapredators do not influence biotic resistance, if any. The mesh size of the cage excluded predators greater than 1 cm in size; therefore, smaller consumers may have foraged on ascidian recruits, bryozoans, and hydrozoans (Collin & Johnson, 2014; Nydam & Stachowicz, 2007). We indeed observed small predators in the nekton (e.g., *Atherina presbyter*,* Gobiusculus flavescens*) as well as among macroinvertebrates, mainly annelids and crustaceans, associated with the fouling community (e.g., *Harmothoe impar*,* Phtisica marina*,* Eualus cranchii*,* Pilumnus hirtellus*). Caging had no effect on mobile fauna (MF) richness, abundance, or assemblage structure. The absence of a cage effect demonstrates that megapredators do not select and forage significantly upon these macroinvertebrates. Furthermore, this result suggests that the cage effect observed on sessile fauna (SF) was not related to any confounding facilitation by macropredators (Lavender et al., 2014; Steele, 1996), supporting our conclusion that megapredators do not affect the studied fouling communities. This negligible predation was unexpected for two reasons. Firstly, our study was performed in late spring–early summer when fish are particularly thought to forage inshore (e.g., *Dicentrarchus labrax*; Pawson, Pickett, Leballeur, Brown, & Fritsch, 2007). Secondly, although the effects of swimming megapredators upon pontoon communities tend to be mixed in the literature (e.g., Connell, 2001a; Lavender et al., 2014), null effects have virtually never been reported. One possible explanation is that these predators are transient and use this shaded habitat during the daytime (when abundance is usually assessed), but preferentially forage in other habitats (Oricchio et al., 2016) at night.

In aquatic systems (see Kovalenko et al., 2012 for review), habitat complexity is often assumed—in addition to increasing microhabitat (physical niche) diversity—to protect organisms from environmental stresses (e.g., water motion, sand‐scouring), to promote organic matter retention, and to mediate biological interactions (e.g., predation). Enhancing complexity of artificial structures may therefore be a pivotal eco‐engineering strategy for maintaining biodiversity in response to marine urbanization (Dafforn et al., 2015; Firth et al., 2016; Loke et al., 2015). However, in our study, modifying the initial habitat attributes did not modify species diversity or assemblage structure in the studied marinas. In particular, despite high MF abundance and richness revealed in this study, turf density—which affects both complexity and the surface area (Tokeshi & Arakaki, 2012)—did not influence their diversity, abundance, and assemblage structure. Relationships between habitat complexity and MF assemblages have been previously supported by similar experimental approaches in aquatic habitats (reviewed in Kovalenko et al., 2012). However, responses may vary with the local species pool as well as abiotic and biotic stressors (Matias, 2013; Strain et al., in press). For example, by manipulating either the size spectrum or the type of structural elements, and controlling for the surface area along intertidal seawalls, Loke and Todd (2016) observed a positive relationship between habitat complexity and diversity (species richness and assemblage structure) at low, but not at high, heights on the shore. Floating pontoons are unique habitats that have specific abiotic and biotic stressors (Piola & Johnston, 2008; Rogers et al., 2016). For instance, they are unlikely to experience sand‐scouring owing to their distance from the bottom (Holloway & Connell, 2002), especially within enclosed marinas (Floerl & Inglis, 2003; Rivero et al., 2013). Although microhabitat complexity may have protected organisms from some important stressors in other systems (e.g., desiccation in the intertidal; Strain et al., in press), it perhaps did not efficiently prevent the occurrence of other important stressors under pontoons, such as exposure to pollutants (Saloni & Crowe, 2015). Considering the negligible effect of megapredators revealed herein, a limited influence of physical stresses (hydrodynamics, scouring) may explain why the initial “complexity” and “exclusion” had no interactive effects on species diversity and distribution in our study.

Interestingly, although artificial complexity had no effect on associated assemblages, we observed an important effect of the sessile fouling communities that grew on the panels during the course of the experiment. This effect seems to be driven by the spatial arrangement of habitat‐engineering species such as solitary ascidians as well as the associated SF. This result echoes an experiment carried out in Australia by Smith, Johnston, and Clark (2014): Using plastic panels on which they observed an effect of panel groove density on SF diversity and assemblage structure after 1 month, the effect of the initial complexity could no longer be detected after 3 months. This loss in initial complexity was attributed to the development of a “*secondary biotic complexity*” (Smith et al., 2014). Similarly, our turf mimics were often completely enveloped by the tunics of colonial and solitary ascidians by the end of the trial. Most of the initial interstitial surfaces within and among turfs were replaced by the interstitial surfaces created with the development of SF. Therefore, modifying any other variable of the initial artificial complexity (e.g., number, size range, relative abundance, and spatial arrangement of structural elements; Tokeshi & Arakaki, 2012; Loke et al., 2015) would likely lead to similar results. Although each sessile species may differ in its habitat‐forming traits (e.g., surface area, shape, texture, chemical and behavioral deterrents), SF generally provide associated epibionts with “*at least as much settlement surface as they occupy*” (Sellheim et al., 2010). The overall interstitial volume (ecospace) is likely to capture the majority of habitat‐forming traits, irrespective of species‐specific associations between SF and MF. This property may explain why ecospace was relatively well correlated with overall MF richness and abundances in both sites. The dominant MF were tube‐dwelling amphipods (*Jassa*,* Monocorophium*,* Apocorophium*), likely benefiting from the surface created and sediment retained within interstices (Sellheim et al., 2010). In addition to partitioning physical niches (size range, specific habitat associations), interstitial diversity and differential sediment accumulations also likely contributed to promoting richness across MF taxa (here dominated by 54 Arthropoda, 49 Annelida, and 22 Mollusca). Although estimating the total volume of interstices provides important insights into the role of habitat‐formers in structuring mobile faunal assemblages, it does not capture the actual complexity of the habitat (no more than any metric; Tokeshi & Arakaki, 2012). Mobile fauna herein varied significantly with the interstitial volume as well as the sessile fauna community structure, suggesting that further investigation regarding habitat–diversity relationship in artificial structures is needed to understand all underlying mechanisms.

Within fouling communities, sessile NIS were abundant and accounted for a large proportion of SF richness, as typically reported across floating artificial habitats (Mineur et al., 2012) including in our study area (Bouchemousse, 2015). In contrast, MF richness was dominated by native species. Most of the observed MF species are important components of seaweed‐associated communities (turf‐forming understory) dominating natural rocky reefs in the region, and within which NIS are virtually absent (Leclerc et al., 2015, 2016; Schaal, Leclerc, Droual, Leroux, & Riera, 2016). The richness and structure of these assemblages were independent of the factor “complexity” which mimicked one of their natural habitats (thick thallus turfs; Connell et al., 2014). By modifying habitat properties, SF assemblages, which are made of large proportions of NIS, attracted native mobile species from adjacent natural habitats. The more diverse these native MF assemblages are, the more likely they are to include NIS‐specific predators and competitors (i.e., using the same resource: physical and trophic niches). Native MF may therefore contribute locally to “biotic resistance” under floating pontoons (Elton, 1958; Stachowicz et al., 2002). These mobile invertebrates may also colonize the hulls of surrounding ships (Mineur et al., 2012). In this context, the cryptogenic amphipod *Monocorophium acherusicum* displayed a positive relationship between its abundance and ecospace at both sites (Brest: *R *=* *.348, *p *=* *.038; Trébeurden: *R *=* *.372, *p *=* *.026). Habitat facilitation due to sessile assemblages on floating surfaces (hulls, pontoons, aquaculture facilities) may explain why this euryhaline tube‐dwelling species has successfully invaded sheltered subtidal habitats worldwide (Bousfield & Hoover, 1997; Pagad et al., 2016). This simple explanation does not hold for every species. Unlike native members of its family, the nonindigenous amphipod *Aoroides longimerus* does not dwell within interstices, but loosely attaches to and swims upon the surface of various biogenic structures (Gouillieux et al., 2016). Although its specific niche may have weakened competitive exclusion at the study sites, this feature may also explain why its abundance did not vary with ecospace (*R *=* *.076, *p *=* *.657). Our results corroborate the importance of floating structures, such as hulls and pontoons as, respectively, vectors and corridors of novel introductions of SF and MF in coastal areas (Mineur et al., 2012).

Our study suggests that under low consumer pressure, mobile (and probably sessile) assemblages on floating pontoons are mainly driven by biotic habitat formation rather than by physical structure through time. This outcome echoes previous results (e.g., Rius, Branch, Griffiths, & Turon, 2010; Sellheim et al., 2010; Smith et al., 2014). The correlations between ecospace and MF categories appeared site‐dependent (Figure 4), which may be due to different species pools and relative abundances between sites. Furthermore, the significant relationships between SF and MF community structure suggest that spatial arrangement, dominance, and species composition all matter. Given that stronger relationships were observed when considering all SF (solitary tunicates and associated species), habitat‐forming traits are likely to be pervasive within fouling communities. At an early stage of successional development, the surface occupancy of long‐lived habitat‐formers such as mussels was negligible on our experimental panels. Only *Mytilus* settlers and juveniles were observed. However, considering their abundances underneath pontoons (JCL, pers. obs.), these habitat‐formers may play an important role in older assemblages. Our short‐term experiment does not take into account the natural fluctuations in recruitment and abundance of habitat‐builders in marinas. Context dependency is a common feature of experimental studies (O'Connor & Crowe, 2005), and modifying either the timing or the duration of the experiment may have potentially led to conflicting conclusions. Although important biotic interactions take place within a few weeks or months and provide important insights to the ecology of fouling communities (Lord & Whitlatch, 2015; Sellheim et al., 2010; Stachowicz et al., 2002), the dynamics of these systems over longer periods (across seasons and years) are required before drawing any hard conclusions (Sutherland & Karlson, 1977), particularly those for designing ecological engineering applications (Bell, Middlebrooks, & Hall, 2014; Chapman & Underwood, 2011; Dafforn, 2017). However, the disturbance regime resulting from maintenance work performed on pontoons is high and natural environmental disturbances can also dramatically change the fouling community structure (e.g., massive die‐offs of habitat‐formers due to rainfall; Pineda et al., 2016; Bouchemousse et al., 2017). Disturbance and biotic and abiotic stresses may influence both the diversity and the nature of species interactions throughout succession (Bennett et al., 2015; Bertness & Callaway, 1994; Sousa, 1979): The facilitation here documented, at an early stage of development and under low consumer pressure, is thus nevertheless important to consider in these particular habitats (Bulleri, Benedetti‐Cecchi, Jaklin, & Iveša, 2016; Dafforn et al., 2015; Strain et al., in press). The continuation of facilitation throughout community development deserves further investigation, because theoretical studies suggest that it can substantially affect the outcomes of biotic resistance processes (Bulleri et al., 2008). The potential cascade of these complex interactions under higher predation pressure (on either SF or MF) or other stresses also deserves close attention (Freestone, Ruiz, & Torchin, 2013; Thomsen et al., 2010).

## 5 CONCLUSIONS

In two marinas with different characteristics, we demonstrated the prevalence of biotic habitat formation over artificial habitat complexity in shaping fouling communities at an early stage of development, under low predation pressure. Sessile fauna that settled and grew on the experimental panels provided new space to be colonized by diverse and abundant mobile fauna, likely immigrants from adjacent natural habitats. Our study suggests that incorporating artificial complexity beneath floating pontoons—one of the approaches used in ecological engineering to promote biotic resistance in particular—may be ineffective: Artificial complexity is likely to be rapidly overwhelmed by habitat engineering within the fouling assemblages, at least under low predation pressure. Altogether, our results suggest that, on the one hand, sessile assemblages can attract diverse and abundant mobile native species and thus increase biotic resistance, and on the other hand, sessile fauna, composed of many NIS, may facilitate the establishment of other NIS and promote their spread due to the presence of ships in the vicinity. Further investigation is required to better determine the balance between biotic resistance and facilitation processes due to these habitat‐forming species, particularly to examine the extent of changes in habitat‐forming traits over succession in fouling communities.

## DATA ACCESSIBILITY

Data available from the Dryad Digital Repository: https://doi.org/10.5061/dryad.80780

## CONFLICT OF INTEREST

None declared.

## AUTHOR CONTRIBUTIONS

JCL and FV conceived the ideas and designed methodology. JCL collected and analyzed the data. JCL and FV collectively wrote the manuscript.
