# Supplementary material for: Habitat formation prevails over predation in influencing fouling communities
Source: Ecol Evol. 2017 Nov 30;8(1):477–92. doi: 10.1002/ece3.3654 (PMC5756867; doi:10.1002/ece3.3654)
Supplement: Supplementary file 1 [file ECE3-8-477-s001.docx]

**Appendix S1.** Assessment of mobile megafauna abundances in July 2014 (i.e. mid-experiment time) along floating pontoons and backward boulders. *This appendix includes Fig. S1, Fig. S2 and Table S1.*

**Pontoon habitat**

*Methods:*

Megafaunal organisms were surveyed along and below 4 parallel pontoons evenly distributed across the harbour. The survey was carried out at mid-experiment time (July 2014). All assessments were performed by the same diver (JCL).

Sampling Unit = 6 x [20 m transect (2-3’) + 3’ stop] below each pontoon

Variable recorded: Species abundance (individual counts and 50 individual increments for small clustered fish shoals, e.g. *Gobiusculus flavescens*, *Atherina presbyter,* complete list and species authorities at the end of this appendix)

Univariate (abundance) analysis: Euclidean distance matrices generated from untransformed abundance data as no transformation permitted homoscedasticity ([Underwood, 1997](#_ENREF_1))

Multivariate ordination: Bray Curtis similarity matrices generated from fourth root-transformed abundance data (with a dummy variable to account for empty units)

*Results*:

Megafauna was dominated by carnivorous and omnivorous species. Although the abundance and multivariate assemblage structure of megafauna were different between sites (Table S1), the most abundant species were encountered in both and their total abundances were similar. The main difference between sites was due to contrasting abundances observed within *Gobiusculus flavescens* shoals, while abundances of large fish, such as *Chelon labrosus*, was overall comparable. Finally it is noteworthy that the distribution of megafauna is fairly homogenous within the harbour (c.f. “Pontoon” term, *P* = 0.06 in Table S1) Dispersion was however heterogeneous at pontoon scale for both abundances and multivariate assemblage structure (Table S1).

**Table S1**. Results of PERMANOVA tests for differences in megafauna abundances and multivariate assemblage structure along floating pontoons. PERMDISP tests for differences in multivariate dispersion around the centroid among nested factors are summarised (NS: p > 0.05, *: p < 0.05, **: p < 0.01, ***: p < 0.001).

| Megafauna | | Abundances | |  |  |  | Abundance distributions | | |  |
| --- | --- | --- | --- | --- | --- | --- | --- | --- | --- | --- |
| Transformation | | None |  |  |  |  | Fourth root | |  |  |
| Distance/Similarity | | Euclidean distance | | |  |  | Bray-Curtis similarity | | |  |
| Source | df | DISP | MS | Pseudo-*F* | *P* |  | DISP | MS | Pseudo-*F* | *P* |
| Site (Si) | 1 | ** | 70917 | 4.612 | 0.059 |  | NS | 12615 | 9.955 | **0.028** |
| Pontoon (Si) | 6 | ** | 15378 | 1.839 | 0.101 |  | * | 1267.2 | 1.708 | 0.058 |
| Res | 40 |  | 8361.2 |  |  |  |  | 742.13 |  |  |


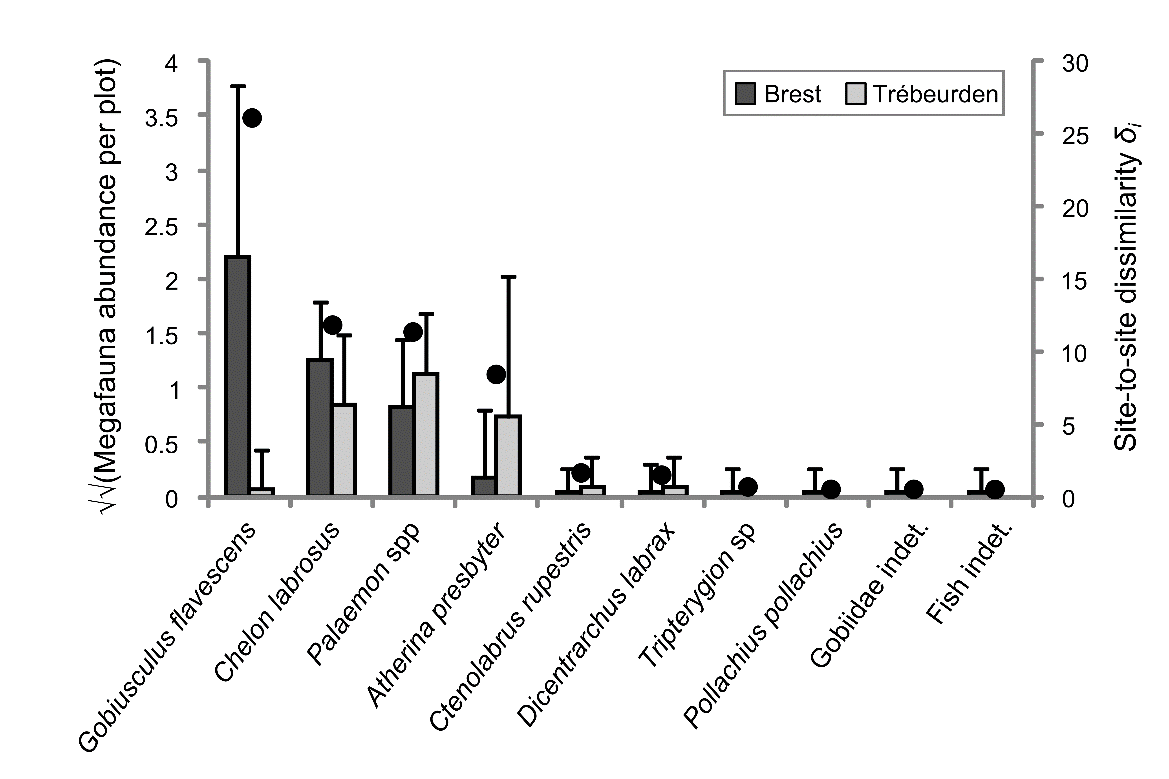


**Figure S1**. Abundances of megafauna (per sampling unit, 20 m transect (2-3’) + 3’ stop) along floating pontoons and average site-to-site contribution to dissimilarity (Circles) in assemblage structure (SIMPER, cut-off 90% of total dissimilarity).

**Boulder habitat (Inner part of the marina)**

*Methods:*

Assessed within 6 plots (field of vision) 20 m apart along boulders (riprap) located nearby the pontoons where experimental panels were deployed.

Survey done at mid-experiment time (July 2014). All assessments were performed by the same diver (JCL).

Sampling Unit = 3’ stop within 6 plots 20 m apart

Variable recorded: Species abundance (individual counts and 50 individual increments for small clustered fish shoals, e.g. *G. flavescens*, *A. presbyter*). Only highly mobile megafauna, i.e. susceptible to move from this habitat to pontoons (e.g. excluding limpets) were considered.

Univariate (abundance) analysis: Euclidean distance matrices generated from untransformed abundance data as no transformation permitted homoscedasticity ([Underwood, 1997](#_ENREF_1))

Multivariate ordination: Bray Curtis similarity matrices generated from fourth root-transformed abundance data (with a dummy variable to account for empty units)

*Results*:

Dominated by carnivores and omnivores, the megafauna encountered is likely restricted to shallow parts of the harbours, though representing an important component of the harbour communities. Like along pontoons, total abundances were similar between sites (PERMANOVA: *F*_1,10_ = 1.101, *P*(perm) = 0.343) but significant differences in multivariate assemblage structure were observed (*F*_1,10_ = 13.442, *P*(perm) = 0.002); these differences are mainly due to *Gobiusclus flavescens* shoals (20.4 %*δi*)*.*


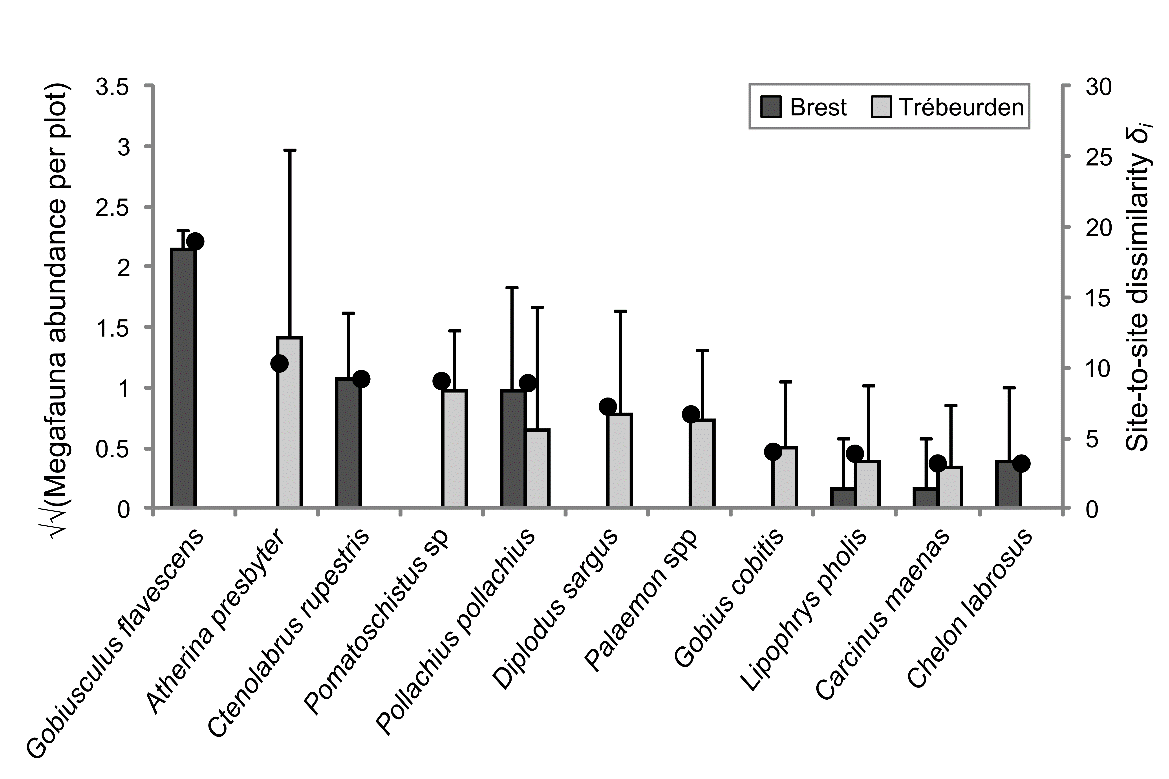


**Figure S2.** Abundances of megafauna (per sampling unit, 6 x 3’ stop on plots 20 m apart) nearby boulders in the inner part of studied marinas and average site-to-site contribution to dissimilarity (Circles) in community structure (SIMPER, cut-off 90% of total dissimilarity).

**Full list of megafauna observed during the survey**

The main feeding regime is indicated in brackets (C: Carnivore, O: Omnivore, U: Unknown)

**Perciformes**

*Chelon labrosus* Risso (O)

*Dicentrarchus labrax* L. (C)

*Diplodus sargus sargus* L. (C)

*Lipophrys pholis* L. (C)

*Centrolabrus exoletus* L. (C)

*Ctenolabrus rupestris* L. (O)

*Tripterygion* Risso (C)

*Gobius cobitis* Pallas (C)

*Gobius paganellus* L. (C)

*Gobius niger* L. (C)

*Gobiusculus flavescens* Fabricius (C)

*Pomatoschistus* Gill (C)

Gobiidae indet. (U)

**Atheriniformes**

*Atherina presbyter* Cuvier (C)

***Gadiformes***

*Pollachius pollachius* L. (C)

Fish indet. (U)

**Decapoda**

*Palaemon* Weber (C)

*Carcinus maenas* L. (C)

**References**

Underwood, A.J. (1997) *Experiments in ecology: their logical design and interpretation using analysis of variance*. Cambridge University Press.
